# Smoking-induced subgingival dysbiosis precedes clinical signs of periodontal disease

**DOI:** 10.1038/s41598-023-30203-z

**Published:** 2023-03-07

**Authors:** Ryan Tamashiro, Leah Strange, Kristin Schnackenberg, Janelle Santos, Hana Gadalla, Lisa Zhao, Eric C. Li, Emilie Hill, Brett Hill, Gurjit S. Sidhu, Mariana Kirst, Clay Walker, Gary P. Wang

**Affiliations:** 1 Department of Medicine, Division of Infectious Diseases and Global Medicine, College of Medicine, University of Florida, Gainesville, FL USA; 2 Department of Periodontology, College of Dentistry, University of Florida, Gainesville, FL USA; 3 Department of Oral Biology, College of Dentistry, University of Florida, Gainesville, FL USA; 4 Department of Endotontics, College of Dentistry, University of Florida, Gainesville, FL USA; 5 Medical Service, North Florida/South Georgia Veterans Health System, Gainesville, FL USA

**Keywords:** Clinical microbiology, Microbiome, Clinical microbiology

## Abstract

Smoking accelerates periodontal disease and alters the subgingival microbiome. However, the relationship between smoking-associated subgingival dysbiosis and progression of periodontal disease is not well understood. Here, we sampled 233 subgingival sites longitudinally from 8 smokers and 9 non-smokers over 6–12 months, analyzing 804 subgingival plaque samples using 16 rRNA sequencing. At equal probing depths, the microbial richness and diversity of the subgingival microbiome was higher in smokers compared to non-smokers, but these differences decreased as probing depths increased. The overall subgingival microbiome of smokers differed significantly from non-smokers at equal probing depths, which was characterized by colonization of novel minority microbes and a shift in abundant members of the microbiome to resemble periodontally diseased communities enriched with pathogenic bacteria. Temporal analysis showed that microbiome in shallow sites were less stable than deeper sites, but temporal stability of the microbiome was not significantly affected by smoking status or scaling and root planing. We identified 7 taxa—*Olsenella sp.*, *Streptococcus cristatus, Streptococcus pneumoniae, Streptococcus parasanguinis*, *Prevotella sp., Alloprevotella sp*., and a *Bacteroidales sp.* that were significantly associated with progression of periodontal disease. Taken together, these results suggest that subgingival dysbiosis in smokers precedes clinical signs of periodontal disease, and support the hypothesis that smoking accelerates subgingival dysbiosis to facilitate periodontal disease progression.

## Introduction

Periodontitis is a polymicrobial infection of the gums and teeth-supporting bone affecting nearly 750 million people worldwide^1^. The etiology of periodontitis is multifactorial, but dysbiosis of the subgingival microbiome plays an important role^2–5^. Healthy subgingival space hosts a microbial community generally dominated by *Streptococci* and other commensal organisms^4–7^. In periodontal disease, the subgingival microbiome shifts towards a more diverse community characterized by putative periodontal pathogens and other gram-negative organisms^2,4–7^. Colonization by these organisms elicits an immune response and inflammation, leading to destruction of the surrounding tissue and tooth loss if left untreated^3^. While poor oral hygiene is a major contributor, systemic diseases and other behavioral lifestyle may also disrupt the ecosystem equilibrium, leading to subgingival dysbiosis^8–10^.

Cigarette smoking has wide ranging adverse health consequences. Smokers are at least 50% more likely to develop periodontal disease than non-smokers, and smoking is associated with disease severity in a dose-dependent manner^11,12^. Smoking drastically alters the microbial ecology of the oral cavity through nutrient deprivation, impairment of the immune system, oxygen depletion, and anti-microbial effects^13,14^, thereby shifting the subgingival microbial composition and structure. Cigarette smoke increases the diversity of subgingival communities, reduces the abundance of beneficial bacteria, and favors colonization of pathogenic species^10,15–20^. Thus, smoking may accelerate the development of periodontal disease by altering the subgingival microbiome.

Subgingival microbiome associated with and without periodontal disease in smokers have been investigated in several studies^10,15–20^. However, the longitudinal nature of the subgingival microbiome has not been well characterized. Most published studies have been cross-sectional^4–6^, and thus the organisms associated with clinical progression of periodontal disease in smokers have not been clearly defined. In the present study, we analyzed 804 subgingival plaque samples from 233 unique subgingival sites from 8 smokers and 9 non-smokers at 3–4 time points over 6–12 months. This large longitudinal dataset allowed us to compare and contrast the temporal dynamics of subgingival microbiome in smokers and non-smokers, and identify microbes associated with progression of periodontal disease. To our knowledge, this is one of the most extensive survey of longitudinal subgingival microbiome in smokers to date.

## Results

Baseline characteristics of 8 smokers and 9 non-smokers are shown in Table 1. The average mean probing depth, clinical attachment loss, and plaque index were higher in smokers compared to non-smokers (3.88 vs. 3.17; 3.58 vs. 1.49; 0.926 vs. 0.468, respectively). For each subject, subgingival plaque samples from the same sites were collected 3–4 times over 6 to 12 months. A total of 804 samples were sequenced, generating 20,030,627 reads with a mean of 24,914 reads per sample (range = 1978–742,281 reads per sample). We identified 822 unique OTUs belonging to 12 phyla and 185 different genera. Samples from non-smokers were dominated by five phyla: *Firmicutes* (28.4%), *Actinobacteria* (19.5%), *Bacteroidetes* (18.8%), *Fusobacteria* (14.9%) and *Proteobacteria* (14.4%), and the remaining five phyla each comprised less than 4% of the total community. Subgingival microbiome from smokers were dominated by three phyla: *Bacteroidetes* (31.3%), *Fusobacteria* (22.6%), and *Firmicutes* (20.4%). For the subsequent alpha and beta diversity analysis, samples were rarefied to 8000 reads, which excluded 28 (3.5%) of 804 total samples due to shallow sequencing depths.

**Table 1:** Baseline characteristics of study participants.

Subject	Age	Sex	Stage	Smoking status	Number of time points (month sampled)	Number of sites	Probing depth (mm)	Clinical attachment loss (mm)	Plaque index	Proportion of samples with probing depth > 3 mm (%)
AB	30	F	I	Non-smoker	4 (0/3/6/12)	16	2.6 ± 0.5	0.0 ± 0.0	0.1 ± 0.3	2%
AC	35	M	I	Non-smoker	3 (0/3/6)	13	3.0 ± 0.4	0.1 ± 0.2	0.3 ± 0.7	5%
AD	61	M	III	Non-smoker	4 (0/3/7/14)	7	3.9 ± 1.2	4.5 ± 3.8	0.3 ± 0.5	50%
AH	34	M	I	Non-smoker	4 (0/3/6/12)	12	3.1 ± 0.5	0.0 ± 0.2	0.5 ± 0.6	17%
AJ	41	F	I	Non-smoker	4 (0/3/6/12)	16	3.2 ± 0.6	0.4 ± 0.6	0.9 ± 0.8	25%
AL	66	F	II	Non-smoker	4 (0/3/6/12)	17	3.3 ± 0.8	2.1 ± 1.4	0.8 ± 0.9	30%
AM	43	F	II	Non-smoker	4 (0/3/6/15)	15	3.2 ± 0.7	0.5 ± 0.5	0.8 ± 1.0	33%
AX	21	M	II	Non-smoker	3 (0/6/9)	15	2.7 ± 0.5	1.9 ± 2.4	0.5 ± 0.8	0%
CC	20	M	II	Non-smoker	3 (0/4/8)	15	3.1 ± 0.9	1.0 ± 1.2	0.6 ± 0.9	24%
AA	50	F	III	Smoker	4 (0/?/?/?)	14	3.9 ± 1.1	2.7 ± 2.3	0.6 ± 0.7	60%
AR	52	F	III	Smoker	4 (0/3/6/14)	16	4.4 ± 1.9	3.9 ± 2.2	1.2 ± 0.8	67%
AS	54	F	III	Smoker	4 (0/3/7/13)	9	4.3 ± 1.1	2.9 ± 1.8	1.1 ± 0.8	83%
AT	59	M	III	Smoker	3 (0/4/8)	15	4.9 ± 1.4	5.3 ± 2.2	0.8 ± 0.7	80%
AU	47	M	II	Smoker	4 (0/3/6/12)	10	3.4 ± 1.0	0.7 ± 0.9	0.9 ± 1.0	45%
CE	39	F	I	Smoker	3 (0/5/8)	15	2.1 ± 0.6	0.7 ± 0.6	0.7 ± 0.8	0%
VJ	45	F	III	Smoker	3 (0/4/8)	13	3.4 ± 1.3	4.4 ± 2.4	2.1 ± 0.8	44%
WH	60	M	III	Smoker	3 (0/4/7)	15	4.3 ± 1.1	6.0 ± 2.0	1.0 ± 0.9	84%

The months when samples were taken are shown in parenthesis separated by slashes. For clinical measurements, mean ± standard deviation is shown.

**Figure 1:**
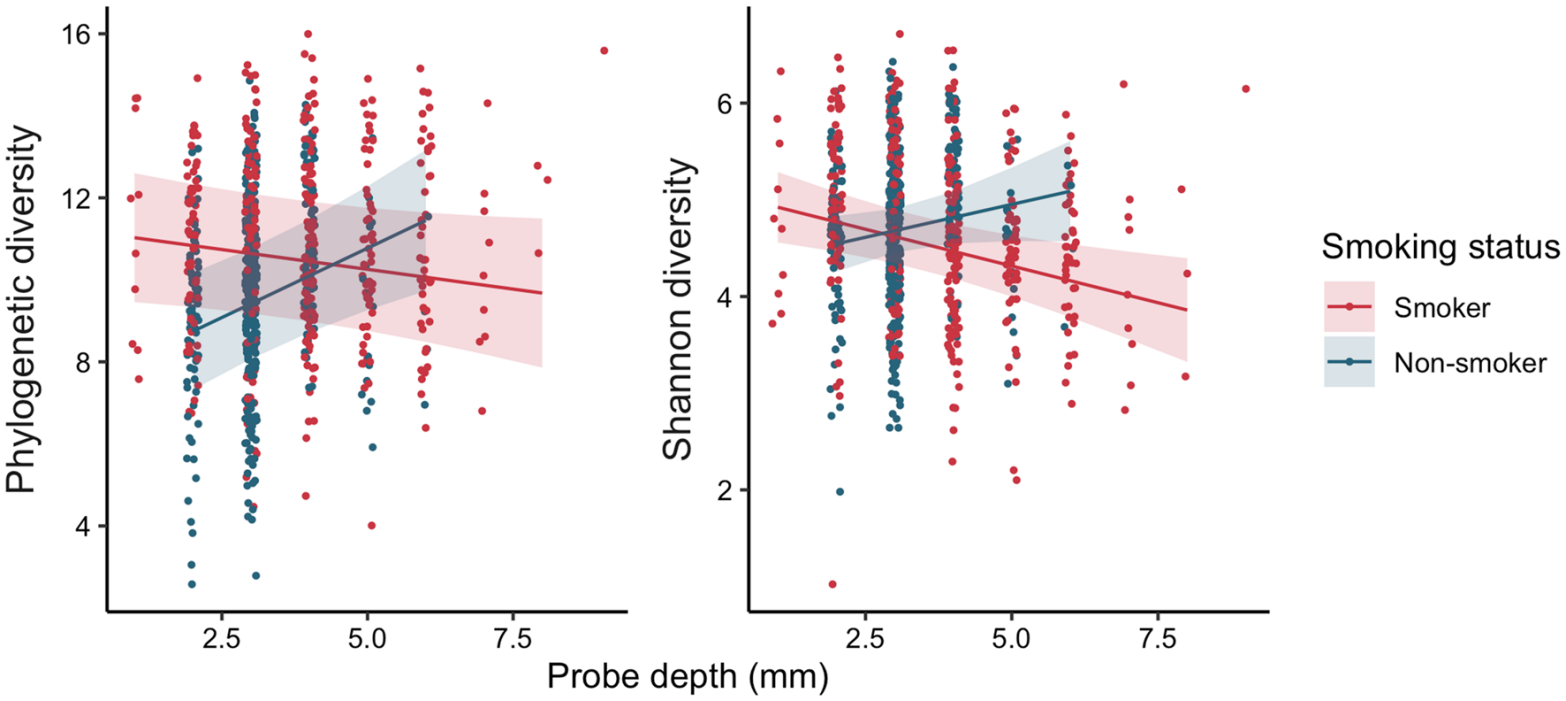
Alpha diversity of subgingival microbiome in smokers and non-smokers according to probing depths. Linear mixed models, which consider the non-independence of samples taken from the same sites and/or at the same time, of Faith’s phylogenetic diversity (Left) and Shannon diversity (Right) are shown as lines with the shaded region representing 95% confidence band of the mean. Each point represents the alpha diversity of a single sample and the color indicates smoking status (red: smoker; blue: non-smoker).

We first examined how probing depth, plaque index, and smoking status influenced the richness (Faith’s phylogenetic diversity) and diversity (Shannon diversity) of the baseline subgingival microbiome using linear mixed models (Fig. 1). With smokers as the reference group, plaque index (b = 0.5682, p = 0.006), smoking status (b = − 3.83, p = 0.005) and the interaction between smoking status and probing depth (b = 0.970, p < 0.001) were significant predictors of phylogenetic diversity while probing depth alone and the interaction between smoking status and plaque index were not. Plaque index (b = 0.255, p = 0.007), smoking status (b = − 0.803, p = 0.028), probing depth (b = − 0.152, p = 0.004) and the interaction between probing depth and smoking status (b = 0.288, p = 0.003) were also significant predictors of Shannon diversity while the interaction between plaque index and smoking status was not. At shallow probing depths, phylogenetic diversity of the subgingival microbiome was higher in smokers compared to non-smokers (Fig. 1). However, smokers and non-smokers were comparable at greater probing depths.

**Figure 2:**
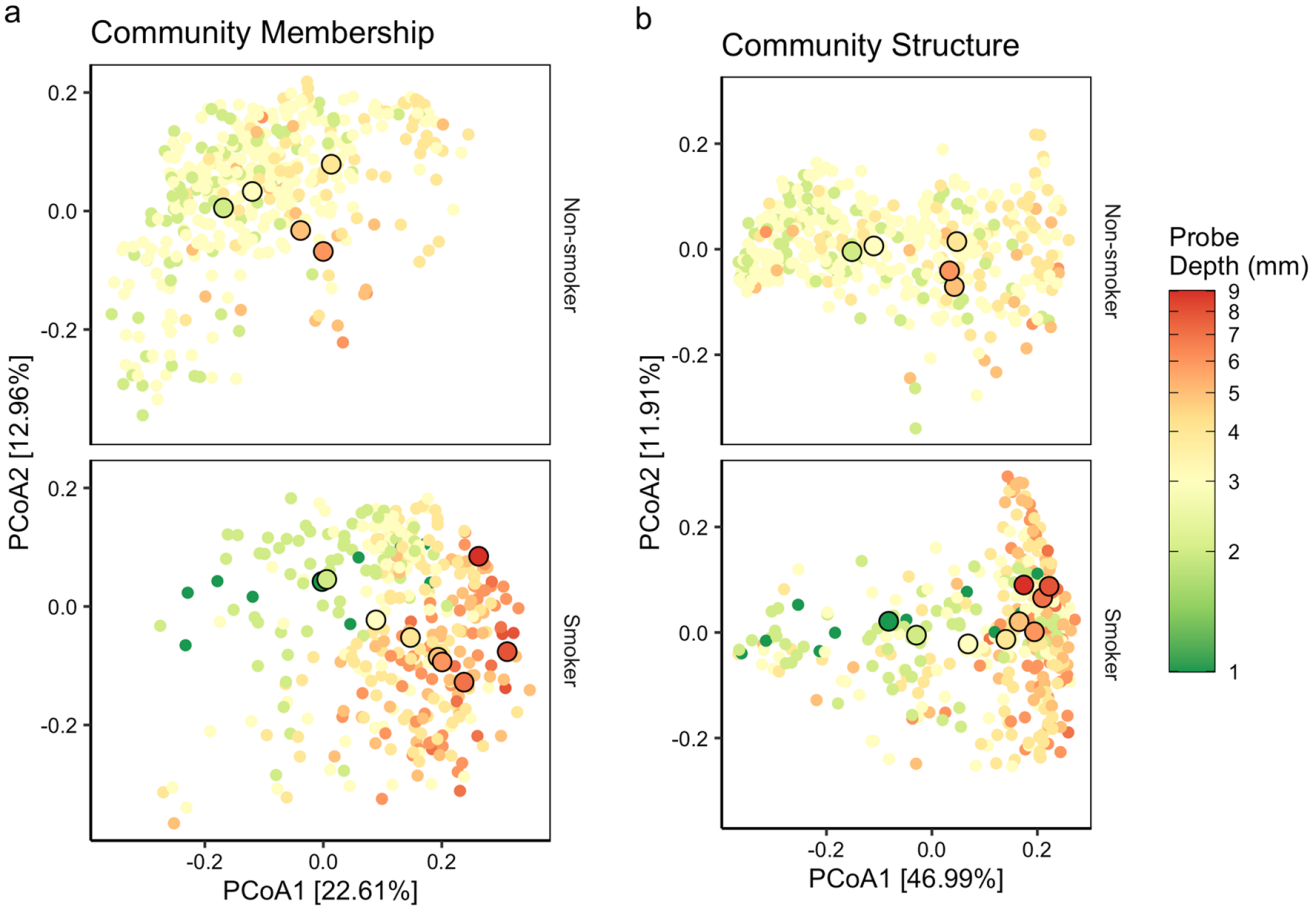
Subgingival microbiomes according probing depth and smoking status based on UniFrac distances. Principal coordinates analysis on (**a**) unweighted and (**b**) weighted UniFrac distances. Non-smokers (top) and smokers (bottom) are showed separately for clarity but are in the same frame for direct comparison. Each point is a single subgingival sample, and the color shows the probing depth of the subgingival site. The centroid for a given probing depth is shown by an outlined circle. For a given probing depth (e.g. green outlined circles), the centroids of smoker sites are shifted to the right compared to the centroids of non-smokers sites. In addition, the centroids of shallow sites of smokers (i.e. green or yellow outlined circles) overlap with the centroids of deeper sites in non-smokers (i.e. orange or red outlined circles), suggesting subgingival dysbiosis in smokers at equal probing depths.

The differences in alpha diversity in shallow sites prompted us to ask whether there were also differences in the overall subgingival microbial communities between smokers and non-smokers. Using weighted and unweighted UniFrac distance metrics, we observed a modest separation between smokers and non-smokers along the first principal axis at equal probing depths from 2 to 5 mm (Fig. 2 and Supplementary Fig. 1). Compared to non-smokers, the subgingival communities in shallow sites of smokers overlapped with communities in deeper sites of non-smokers, indicating dysbiosis or changes in microbial community structure in smokers at equal probing depths. Due to sparse datasets, comparisons between smokers and non-smokers could not be made for 1 mm and ≥ 6 mm sites. The separation between smokers and non-smokers was more pronounced in unweighted UniFrac compared to weighted Unifrac (Fig. 2 and Supplementary Figs. 1 and 2), suggesting a stronger effect of smoking on the minority members of the microbiome. Taken together, these results indicate that, at equal probing depths, the subgingival microbiome of smokers is altered and differs from non-smokers.

To investigate the association between subgingival microbiome, plaque index, probing depth, smoking status, subject identity, and different subgingival sites, we used distance-based redundancy analysis (db-RDA) on both unweighted and weighted UniFrac distances (Table 2). The db-RDA analysis showed that all the variables examined were significantly associated with microbiome differences for both UniFrac metrics. Smoking, plaque index, and probing depth had strong effects on the subgingival microbiome, separating samples almost exclusively along the first db-RDA axis (Supplementary Fig. 3). Overall, the model explained more variation in the community structure than community membership (Table 2), suggesting that the combination of smoking status, probing depth, subject, and site identity had a greater influence on dominant OTUs compared to minority OTUs. Interestingly, we found that subject identity alone explains more variation in community membership than community structure.

**Table 2:** Distance-based redundancy analysis (db-RDA) on UniFrac distances of subgingival microbiome samples.

	Predictors	F statistic	P value	Adjusted R^2^
Community membership (unweighted UniFrac)	Probing depth	51.21	0.001	0.515
	Plaque index	68.32	0.001	
	Smoking status	142.97	0.001	
	Subject identity	26.32	0.001	
	Site identity	2.26	0.001	
Community structure (weighted UniFrac)	Probing depth	53.14	0.001	0.591
	Plaque index	104.25	0.001	
	Smoking status	109.98	0.001	
	Subject identity	14.31	0.001	
	Site identity	1.76	0.001	

db-RDA was constrained by smoking status, probing depth, and subject identity. F statistics and p values were generated through ANOVA like permutation tests using 999 permutations.

**Figure 3:**
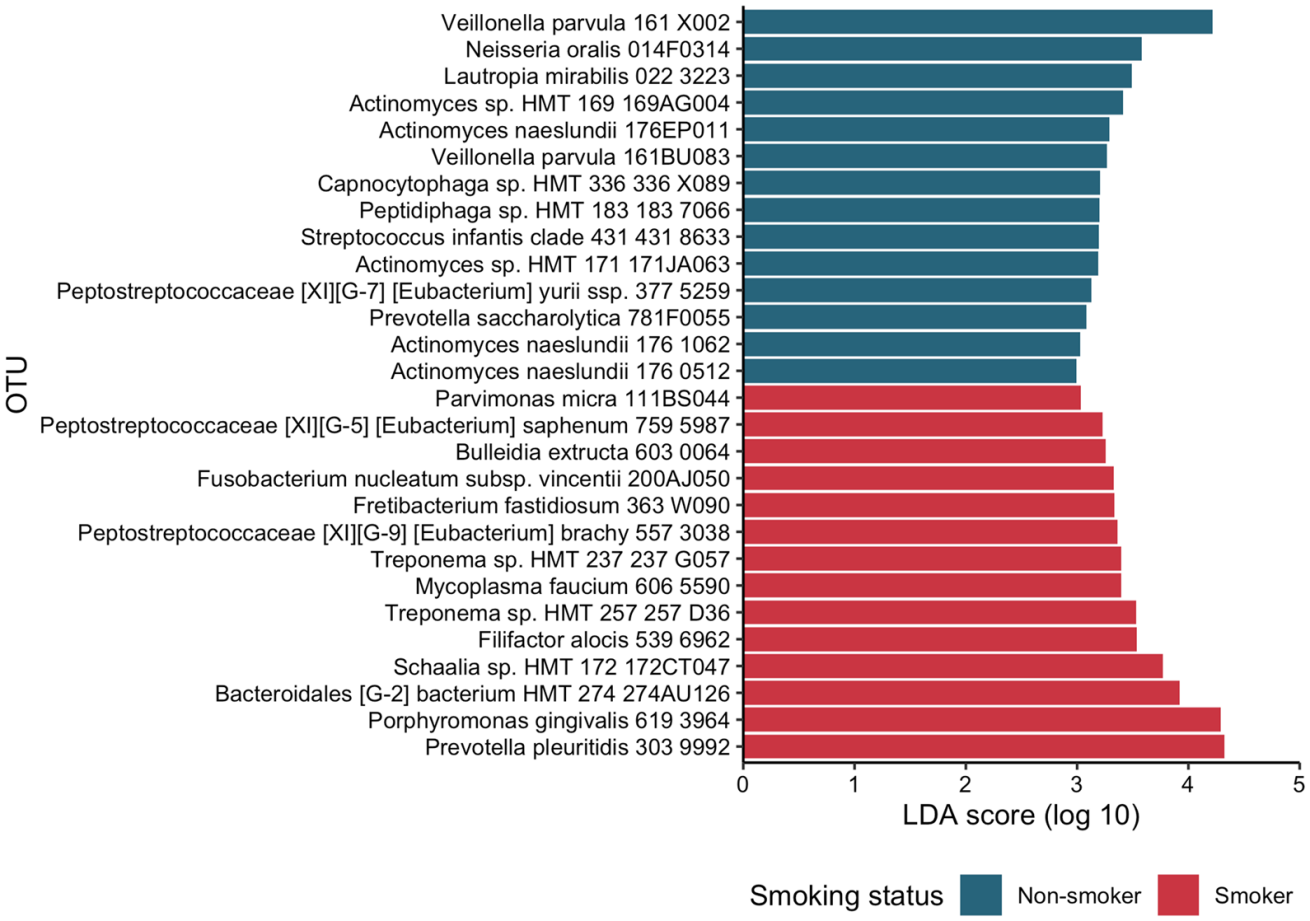
Differentially abundant taxa between smokers and non-smokers. Differentially abundant OTUs were identified using linear discriminant analysis Effect Size (LEfSe). Color indicates the enrichment of the taxa associated with different smoking status. *OTU* operational taxonomic unit.

To identify specific phyla and OTUs associated with altered subgingival microbiome in smokers at the baseline visit, we used the Linear discriminant analysis Effect Size approach (LEfSe). The LEfSe analysis identified *Tenericutes* as the only phyla that were differentially abundant between smokers and non-smokers, which was more abundant in smokers. Other phyla, including *Bacteroidetes*, *Fusobacterium*, *Firmicutes*, and *Actinobacteria*, had larger effect sizes but were not statistically significant. A total of 28 differentially abundant OTUs with an LDA score of greater than 2 were identified (Fig. 3), including 14 OTUs overrepresented in smokers and 14 OTUs more abundant in non-smokers. The association between smoking and the identified taxa was consistent across both shallow (probing depth < 4 mm) and deep (probing depth ≥ 4 mm) sites.

**Figure 4:**
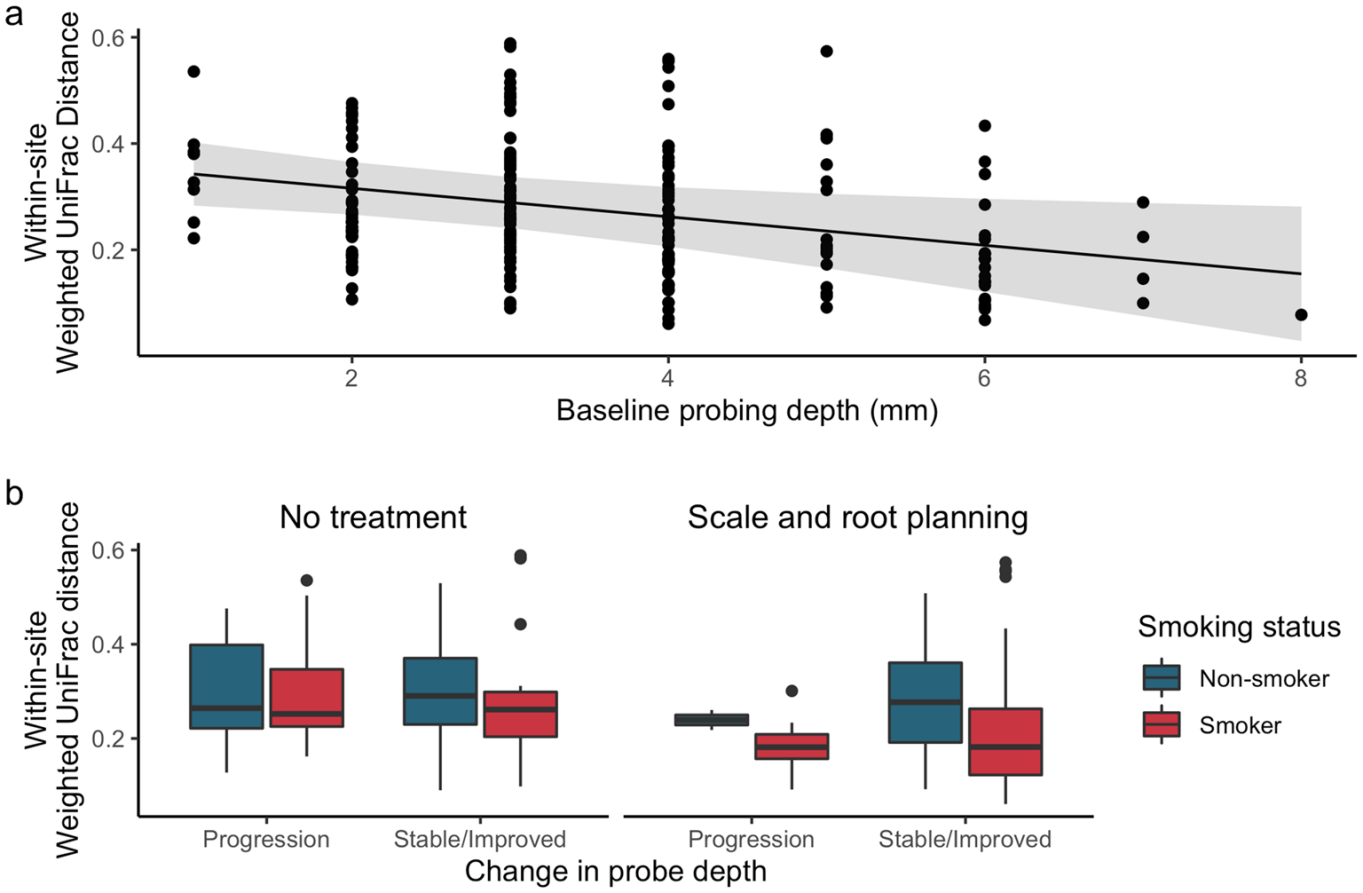
Relationship between smoking status, treatment with scaling and root planing, initial probing depth, and clinical progression on temporal stability of the subgingival microbiome. The impact of (**a**) initial probing depth, and (**b**) smoking status, treatment with SRP, and change in probing depth over time on subgingival microbiome stability was evaluated using mixed linear models. Each point represents the collapsed data of longitudinal samples from a single subgingival site, where lower UniFrac distance (y-axis; determined by collapsing data from the same site) indicates temporal stability. (**a**) Shows the predicted marginal effects (line) with the 95% confidence intervals (shaded region). (**b**) Boxplots are divided by treatment status (top heading), change in probe depth (bottom labels), and smoking status (color). Boxplots show the mean (center black line), 1st and 3rd quartiles. The whiskers extend up to 150% of the interquartile range with points representing outliers of that range.

The longitudinal study design allowed us to examine factors associated with temporal stability of subgingival microbiome. First, the longitudinal data of each subgingival site was collapsed into a single measure by taking the weighted UniFrac distance between subgingival microbiome communities at time 0 and 6 months (within-site weighted UniFrac distance). Using this approach, a small UniFrac distance metric for a given site suggests temporal stability, and a large value indicates more variability. Linear mixed models were then used to assess the effects of baseline probing depth, treatment with scaling and root planing (SRP), smoking, and changes in probing depth (indicating stable or disease progression), on the within-site weighted UniFrac distance (or temporal stability). Subject identity was included as a random effect to account for non-independence of multiple sites sampled from the same individual. Linear mixed model analysis revealed that among the factors examined, only baseline probing depth (b = − 0.027, p = 0.017) was associated with stability of community structure (Fig. 4a). Treatment with SRP (0.029, p = 0.292), smoking status (b = 0.034, p = 0.277), and changes in probing depth (b = − 0.006, p = 0.526) were not (Fig. 4b). Thus, subgingival microbiome in shallow sites were more variable, whereas the microbiome in deep sites were more stable over time. Treatment with SRP, smoking status, and clinical stability did not impact the temporal stability of the subgingival microbiome.

To identify specific taxa associated with changes in probing depths over time, we first classified subgingival sites into three groups based on changes in probing depth over six months: clinically progressing (∆probing depth ≥ 2 mm), clinically stable (− 1 mm ≤ Δprobing depth ≤ 1 mm), and clinically improving (∆probing depth ≤ − 2 mm). The vast majority of 233 sites sampled longitudinally had a baseline probing depth between 2 to 5 mm (Supplementary Fig. 4). Among the 233 sites, 104 sites (44.6%) at baseline probed 4 mm or greater and was treated with SRP, and 129 sites (55.4%) probed 3 mm or less and thus received no treatment. Among the 104 sites treated with SRP, 18 sites (17.3%; 12 from smokers and 6 from non-smokers) improved by 2 mm or greater, 82 sites (78.8%) were stable, and 4 sites (3.8%) progressed by 2 mm or greater. Among the 129 untreated shallow sites, 124 sites (96.1%) were stable, but 5 sites (3.9%) progressed by 2 mm or greater. A total of 7 sites from smokers progressed clinically, which included 3 deep sites that had been treated with SRP and 4 shallow sites that were not treated. Only 2 sites from non-smokers progressed, which included 1 deep site treated with SRP and 1 shallow site that was untreated. Interestingly, the progressing sites in smokers shared very similar community membership and structure at baseline compared to clinically stable sites, and ones that progressed in smokers also shared similar community structure to sites that had deeper probing depths (Supplementary Fig. 5).

**Figure 5:**
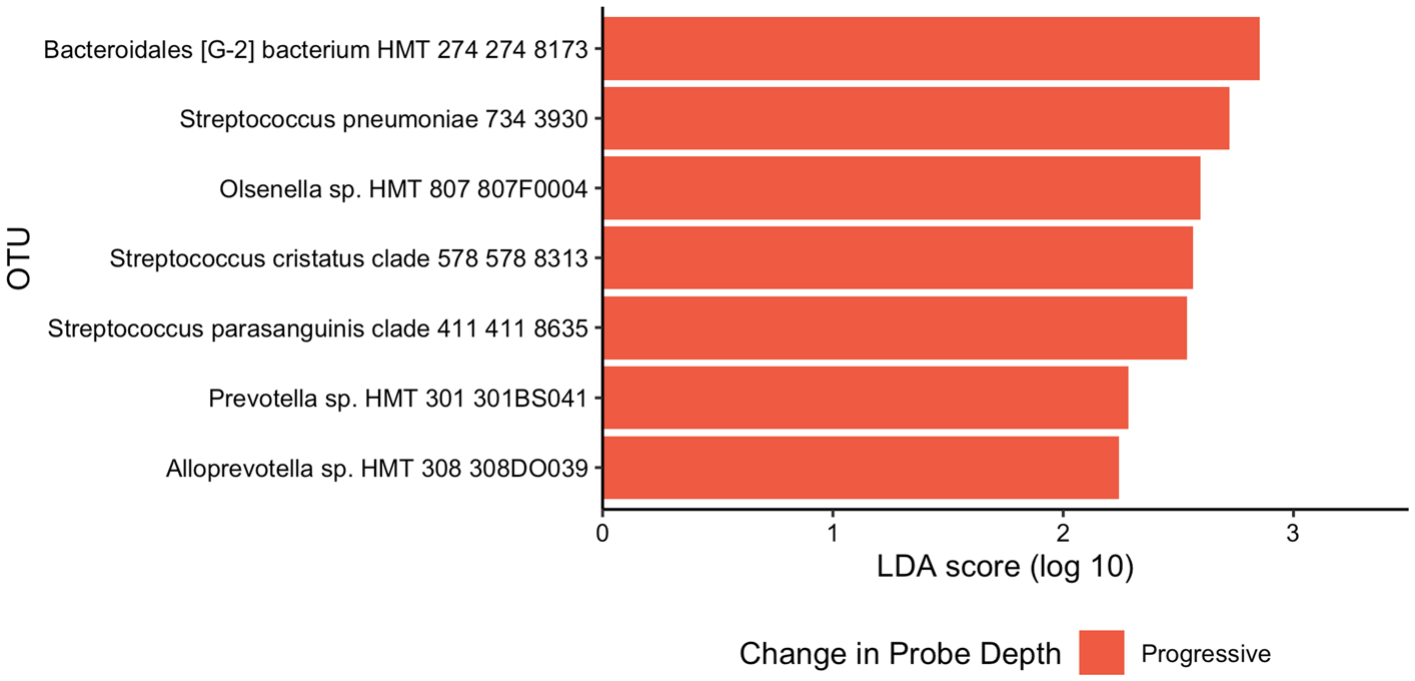
OTUs associated with changes in probing depths determined using linear discriminant analysis Effect Size (LEfSe). Five different subjects were represented in eight progressing sites, with subjects CC, VJ, and WH each contributing two sites. One progressed site was excluded (WH13) due to the lack of a match with a similar (within 1 mm) baseline probing depth in that subject. Color represents enrichment of the OTU associated with changes in probing depths at subgingival sites. OTUs with an LDA score greater than 2 are shown. Sites were classified as progressing if the probing depth increased by 2 mm or more over 6 months. The probing depth for stable sites had no changes over 6 months. Baseline samples of progressing sites were matched to all clinically stable sites with similar (+ /− 1 mm) baseline probing depths within the same subject.

Next, we used LEfSe to compare sites that progressed to sites that were stable by matching progressed sites with stable sites that had similar baseline probing depths (+ /− 1 mm) within each subject. LEfSe analysis identified seven OTUs (*Olsenella sp.*, *Streptococcus cristatus, Streptococcus pneumoniae, Streptococcus parasanguinis*, *Prevotella sp., Alloprevotella sp*., and a *Bacteroidales sp.*) that were enriched in sites associated with clinical progression (Fig. 5). These 7 taxa were not associated with smoking status except for the *Bacteroidales sp.*, which was enriched in smokers (Fig. 3). No OTUs were associated with clinical stability.

## Discussion

The adverse impacts of smoking on human health are well documented^21^. Here, we showed that subgingival dysbiosis is likely another consequence of cigarette smoking. In non-smokers, subgingival microbial communities in shallow sites were considerably less diverse than deep sites. In contrast, shallow sites in smokers had similar diversity as deep sites. Notably, subgingival microbiome in shallow sites of smokers resembled the microbiome dysbiosis in deeper sites of non-smokers. Differential abundance analysis revealed that many taxa associated with smokers have been previously implicated in periodontal disease. However, none of these taxa were associated with clinical progression of periodontal disease. Longitudinal analysis showed that subgingival microbiome in shallow sites were less stable compared to deeper sites, but the temporal variability was not affected by smoking status or scaling and root planing. Taken together, our results support the hypothesis that smoking facilitates the development of subgingival dysbiosis associated with periodontal disease.

Consistent with previous studies, we showed that species richness and diversity differ between smokers and non-smokers in shallow sites^18^ but not in deep sites^17^. The subgingival microbiomes of smokers share many similarities to the communities of periodontally diseased individuals. In most host-associated microbiomes, a reduction in microbial diversity is often associated with disease and dysbiosis^22^, as organisms are lost and key metabolic pathways are disrupted. Subgingival microbiome differs in that periodontal disease is associated with an increase in microbial richness and diversity^4,5,7,19^. As communities with increased diversity tend to withstand environmental perturbations and pathogen invasion^23,24^, the higher microbial diversity in smokers may withstand dental hygiene practices and commensal colonization, thereby facilitating the development of periodontal disease. Similarly, smoking and periodontal disease may have antagonistic effects on community structure where the impact of smoking decreases as subgingival sites deepen. UniFrac analysis showed that subgingivial microbial communities in shallow sites of smokers resembles the communities in deep sites of non-smokers, which was more pronounced in unweighted compared to weighted analysis. These findings suggest a disproportionate impact on minority members of the subgingival microbiome, leading to dysbiotic communities that are resilient and more stable over time.

Microbial biomarkers associated with periodontal disease have been well described. Putative periodontal pathogens include members of the red complex (*Porphyromonas gingivalis*, *Tannerella forsythia*, and *Treponema denticola*) and the orange complex (*Fusobacterium nucleatum*, *Fusobacterium periodonticum*, *Eubacterium nodatum*, *Parviomonas micra*, *Prevotella intermedia*, *Prevotella nigrescens*, and several *Campylobacter* species)^2^. Among them, none were enriched in non-smokers in our study, and *P. gingivalis* and *F. nucleatum* subspecies were differentially more abundant in smokers (Fig. 3). Several organisms have been associated with healthy subgingival microbiome^4,6^, many of which including *Veillonella parvula* were associated with non-smokers in our study. Recent work has implicated putative periodontal pathogens in systemic diseases. For instance, *F. nucleatum* has been associated with colorectal cancer and adverse pregnancy outcomes (reviewed in^25^), and *P. gingivalis* has been associated with different types of cancers (reviewed in^26^), Alzheimer’s disease^27^ and rheumatoid arthritis^28^. Thus, smoking may facilitate or support a microenvironment that favors putative periodontal pathogens, leading to far-reaching effects on the health of the host.

Previous work showed a high degree of inter-individual variations of the healthy subgingival microbiome but relatively low inter-individual variations in the diseased microbiome^6^. Our longitudinal analysis demonstrated that healthy subgingival microbiome was also characterized by high temporal variation, whereas diseased communities were less variable over time. We found that shallow probing depth was associated with high temporal microbiome variation. After accounting for the initial probing depths, smoking status, treatment with scaling and root planning, and changes in probing depth over time were not associated with variation in the microbiome. Interestingly, many of the sites that progressed to disease clustered with deep sites, irrespective of the probing depth at baseline. Thus, these results support the hypothesis that subgingival dysbiosis in smokers precedes clinical signs of periodontal disease, rather than occurring in concert.

Most studies that characterize differences between healthy and diseased subgingival microbiome have been cross-sectional^4–7^. Thus, the causal relationships between microbiome and progression of disease could not be evaluated. Our longitudinal design allowed us to identify specific taxa associated with clinical progression of periodontitis. Despite sampling 233 sites repeatedly from 17 subjects over 6–12 months, only 9 sites progressed by 2 mm or greater. At baseline, these sites varied in probing depths, and some sites progressed from health to disease whereas other sites had periodontal disease at baseline and progressed during the study. We identified seven OTUs associated with progression of periodontal disease. One fell within the genus *Prevotella*, whose members are often associated with periodontitis^2,4,6^. Conversely, *S. cristatus* has been shown to be overabundant in the healthy subgingiva^6,29,30^. *S. cristatus* is a primary adherence point for *F. nucleatum* and has been shown to suppress immune response to *F. nucleatum* infection^31^. *F. nucleatum* can serve as a “bridge species” that aids in the transition from a healthy, commensal-dominated community to a pathogenic one^31,32^. Late colonizers, many of which are pathogenic, cannot incorporate themselves into the subgingival biofilm in the absence of *F. nucleatum*^32^. Thus, a high level of *S. cristatus* may contribute to disease progression through recruitment and maintenance of *F. nucleatum*. We note that due to the small number of sites that progressed, we could not distinguish between markers associated progression from early dysbiosis to periodontitis and markers for progression of periodontal disease severity.

There has been considerable debate as to whether subgingival dysbiosis is a local (site-specific) or a global (whole-mouth) event. Earlier studies argued for local changes^4,33^ but later studies suggested a more global process^5,7,10^. Our extensive sampling approach allowed us to compare deep and shallow sites within individuals, and our results suggest that the discrepancies in the literature may reflect methodological rather than biological differences. For instance, PCoA on weighted UniFrac distances separated samples primarily by probing depth, whereas PCoA on unweighted UniFrac distances separate samples by subject identity and smoking status (Fig. 2). This suggests that periodontal disease is associated with shifts in the overall community structure rather than the presence or absence of certain specific bacteria. Thus, unweighted distances that quantify differences in community membership may be imperfect measures for detecting differences across healthy and diseased sites within an individual, and the results of Ganesan et al.^10^ may reflect a strong subject effect rather than the lack of a disease effect. Abusleme et al.^5^ found that within-subject matched sites that only differed in bleeding on probing did not differ. As a result, probing depth may be a better indicator of subgingival dysbiosis than bleeding on probing. Altogether, subgingival dysbiosis may be site-specific, resulting from local changes in the abundance rather than the presence of different bacteria as the probing depth increases.

This study has several limitations. First, smoking greatly alters the oral environment^13,14^, but whether the microenvironment allow pathogens to outcompete commensals or directly eliminate commensals remains unknown. Second, mechanistic understanding is inherently limited in observational human studies. Third, this study lacked a sufficient number of subgingival sites that progressed clinically, and the sites that progressed primarily came from smokers and were clinically heterogeneous at baseline. Finally, smokers in our study had slightly more advanced stages of periodontitis than non-smokers at baseline, and the lack of long-term follow up and information regarding radiographic bone loss precluded the determination of grading. Future studies will require full-mouth subgingival sampling in a larger number of periodontally healthy smokers and non-smokers with a much longer follow-up to uncover the successional pattern of dysbiosis and the organisms contributing to or initiating the pathogenic process.

Periodontal disease is a major public health concern. Cigarette smoking disrupts the oral environment and pre-disposes individuals to periodontitis through dysbiosis of the subgingival microbiome. Subgingival communities of smokers are diverse, pathogen-rich, and commensal-poor, but have a similar level of temporal variability as non-smokers. Temporal stability of the subgingival microbiome is modulated by periodontal disease severity. Most notably, subgingival dysbiosis in smokers precedes clinical signs of periodontal disease, supporting the hypothesis that smoking creates a microenvironment that promotes the development of subgingival dysbiosis contributing to periodontal disease. Thus, our study underscores the complex nature of subgingival microbiome and its interaction with environmental gradients. The approach described here should facilitate the design of a larger prospective cohort to further elucidate the transition of subgingival microbiome from health to disease.

## Methods

### Subject recruitment and sample collection

For this pilot study, subjects were recruited from the Periodontology Clinic and the DMD Student Dental Clinic at University of Florida College of Dentistry, Gainesville, Florida from March 2012 to April 2013. All subjects were over 18 years of age and had a minimum of 20 natural teeth. Smoking history was obtained by self-report. Smokers were defined as individuals who smoked ≥ 10 cigarettes per day for at least 5 years, and those who have never smoked were non-smokers. Former smokers were not included in this study. Exclusion criteria included diabetes, pregnancy, lactation, systemic antibiotic use within the previous 6 months, periodontal treatment within the previous 12 months, known congenital or acquired immunodeficiency, and use of any immunosuppressive medications.

Clinical measurements (i.e. probing depth, clinical attachment loss, plaque index) were assessed at each visit. At the initial visit, at least 12 subgingival sites were randomly sampled whenever possible and the same sites were sampled again at three and six months. Nine of 17 subjects (53%) had additional plaque samples collected from the same sites at 12 months (Table 1). At baseline, subgingival site that probed 4 mm or greater had scaling and root planing (SRP), but no subjects were re-treated with SRP during the study. Biofilm on the supragingival surface was removed using sterile gauze, and subgingival biofilm was sampled using sterile endodontic paper points. Each sample was transferred to a sterile tube containing storage buffer (MO BIO, Carlsbad, CA), placed immediately on ice, and stored at − 80 °C until DNA extraction. For each subject, subgingival sites (range: 7–17) were sampled at baseline and the same sites were sampled again at three and six months.

### DNA extraction, PCR amplification, and Illumina sequencing

Genomic DNA was extracted using the MO BIO PowerSoil DNA extraction kit (Carlsbad, CA) according to manufacturer’s instructions. For each sample, the V1-V3 hypervariable region of the 16S rRNA gene was amplified using composite 27F (5′ AGAGTTTGATCCTGGCTCAG 3′) and 534R (5′ ATTACCGCGGCTGCTGG 3′) primers. PCR reaction mixtures contained 4 µl of extracted DNA, 100 nM of the forward primer, 100 nM of the reverse primer, and 10 µL of SuperFi PCR master mix (Invitrogen, Carlsbad, CA, USA). 16S rRNA amplicons were analyzed on 1% SYBR Safe agarose gel. Gel slices containing the amplicons were extracted and purified using Qiagen gel extraction kit (Qiagen, Valencia, CA, USA). Purified PCR products were quantified using Qubit HS DNA quantification kit (Invitrogen, Carlsbad, CA, USA) and pooled in equimolar concentration. qPCR was used to quantify the DNA concentration of the pool and prepare the library for sequencing. The use of barcodes allowed for multiplexing and bidirectional sequencing on the Illumina MiSeq platform (Illumina, San Diego, CA, USA).

### Data processing

Paired-end reads of 300 nt each covering the V1–V3 hypervariable region of the 16S rRNA gene were processed using custom scripts written in R^34^. The reads were filtered based on exact matches to the barcode/primer and an average quality score of 30. Samples were de-multiplexed according to the combination of their unique barcodes (4–8 nt long) on each paired end. The barcodes and primers (27F and 534R) were trimmed, and paired-end reads were joined using FLASh^35^, with a minimum overlap of 10 bp, to reconstruct the original contiguous amplicon. Reads were assigned reference OTUs using the Human Oral Microbiome Database (HOMD) version 15.22^36^ and USEARCH alignment with a 97% identity and 80% aligned query threshold. Reads that did not meet the filtering criteria or reference assignment were excluded from subsequent analysis.

OTU tables from multiple sequencing runs were merged, singletons were filtered out, and 11 samples with fewer than 20 total reads were excluded. Relative abundances were calculated from the unrarefied OTU table. The OTU table was then subsampled down to an even sequencing depth of 8000 reads (Supplementary Fig. 6). Alpha diversity and beta diversity metrics were estimated in QIIME2 (version 2018.8, https://qiime2.org/) using the core-metrics-phylogenetic pipeline. Alpha diversity was measured with species richness (Faith’s phylogenetic diversity) and species diversity (Shannon diversity). Community differences between samples were measured using unweighted and weighted UniFrac distances. Temporal stability of the microbial communities was estimated by the weighted UniFrac distance between the baseline and 6 month sample from the same site (within-site UniFrac distance. Sites with greater within-site distance indicate more variability over time, whereas sites with lower distances are more stable.

### Statistical analyses

Statistical analyses were performed in R v.3.4.2^34^unless otherwise noted. Differences in alpha diversity across smoking status and probing depth were analyzed with linear mixed models using the lmer() function in lme4 v.1.1-19^38^, with site identity and time point nested within subject identity as random effects. Marginal effects for linear mixed models were calculated using ggpredict() in the ggeffects package v.0.6.0^39^. Principal coordinates analysis (PCoA) of Unifrac distances were used to examine clustering of samples. Statistical significance of environmental variables were tested with distance-based Redundancy analysis (db-RDA) using the dbrda() function in vegan v.2.5–3^37^ with smoking status, plaque index, probing depth, subject identity, and site identity included as predictors. Linear mixed models were then used to compare temporal stability of the subgingival community structure (within-site UniFrac distance) across smoking status, treatment status, initial probing depth, and change in probing depth over time. Subject identity was included as a random effect in this analysis to account for multiple sites in the same subject. Differentially abundance analysis was conducted on the unrarefied OTU table with LEfSe (Galaxy version 1.0)^40^, excluding samples with fewer than 8000 reads. First, smokers were compared to non-smokers, where probing depth (shallow vs deep sites) and subject identity were accounted for. Then, LEfSe was used to compare clinically progressing sites (∆probing depth ≥ 2) to within-subject matched stable sites (− 1 ≤ ∆probing depth ≤ 1) that were within + /− 1 mm of baseline probing depths. All differentially abundant OTUs met the minimum LDA score of 2.

### Ethics approval and consent to participate

All subjects provided written informed consent for study participation and procedures. The study was approved by the Institutional Review Board at the University of Florida under project #444-2011. All research was performed in accordance with relevant guidelines/regulations and in accordance with the Declaration of Helsinki.

## Supplementary Information

Supplementary Figures.

## Data Availability

All sequence data are available in DANS under https://doi.org/10.5281/zenodo.762681510.5281/zenodo.7626815 .

## Abbreviations

OTU: Operational taxonomic unit
PcoA: Principal coordinates analysis
db-RDA: Distance-based redundancy analysis
LDA: Linear discriminant analysis
