# Supplementary material for: Smoking-induced subgingival dysbiosis precedes clinical signs of periodontal disease
Source: Sci Rep. 2023 Mar 7;13:3755. doi: 10.1038/s41598-023-30203-z (PMC9992395; doi:10.1038/s41598-023-30203-z)
Supplement: Supplementary file 1 — Supplementary Figures. [file 41598_2023_30203_MOESM1_ESM.docx]

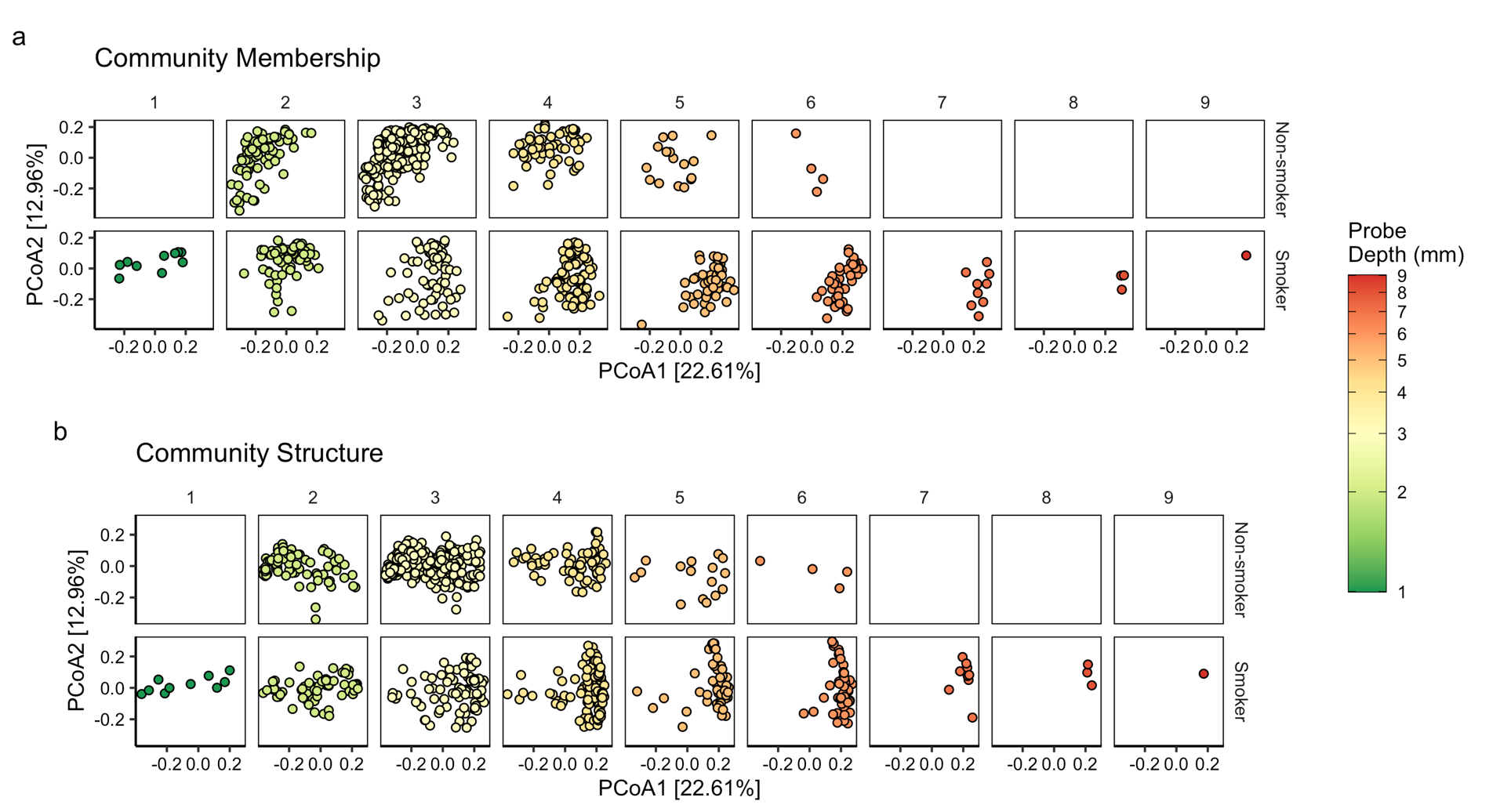


**Supplementary Figure 1:** Subgingival microbiome communities according to probing depths.


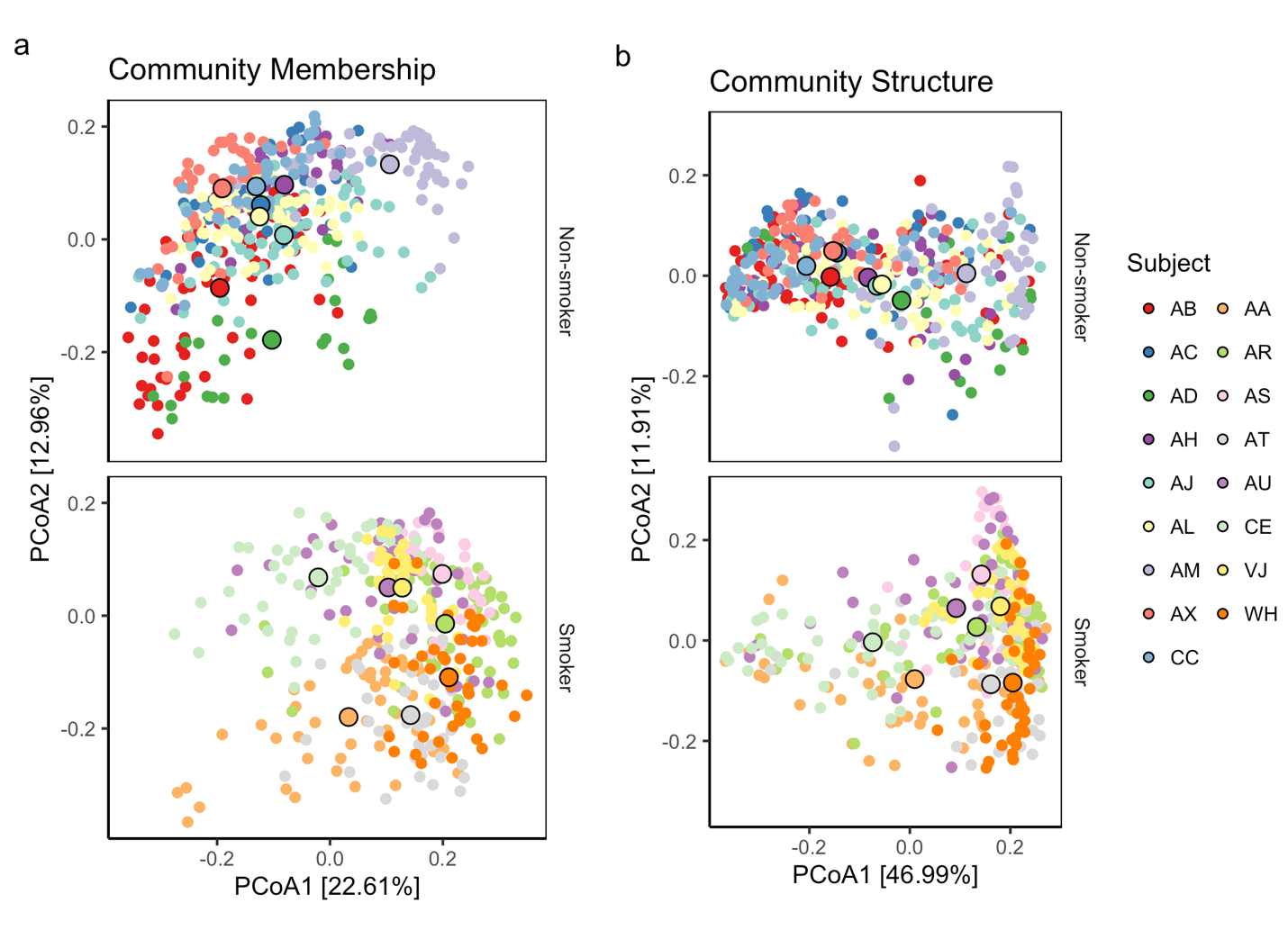


**Supplementary Figure 2**. Subgingival microbiome communities shown by subject identity.


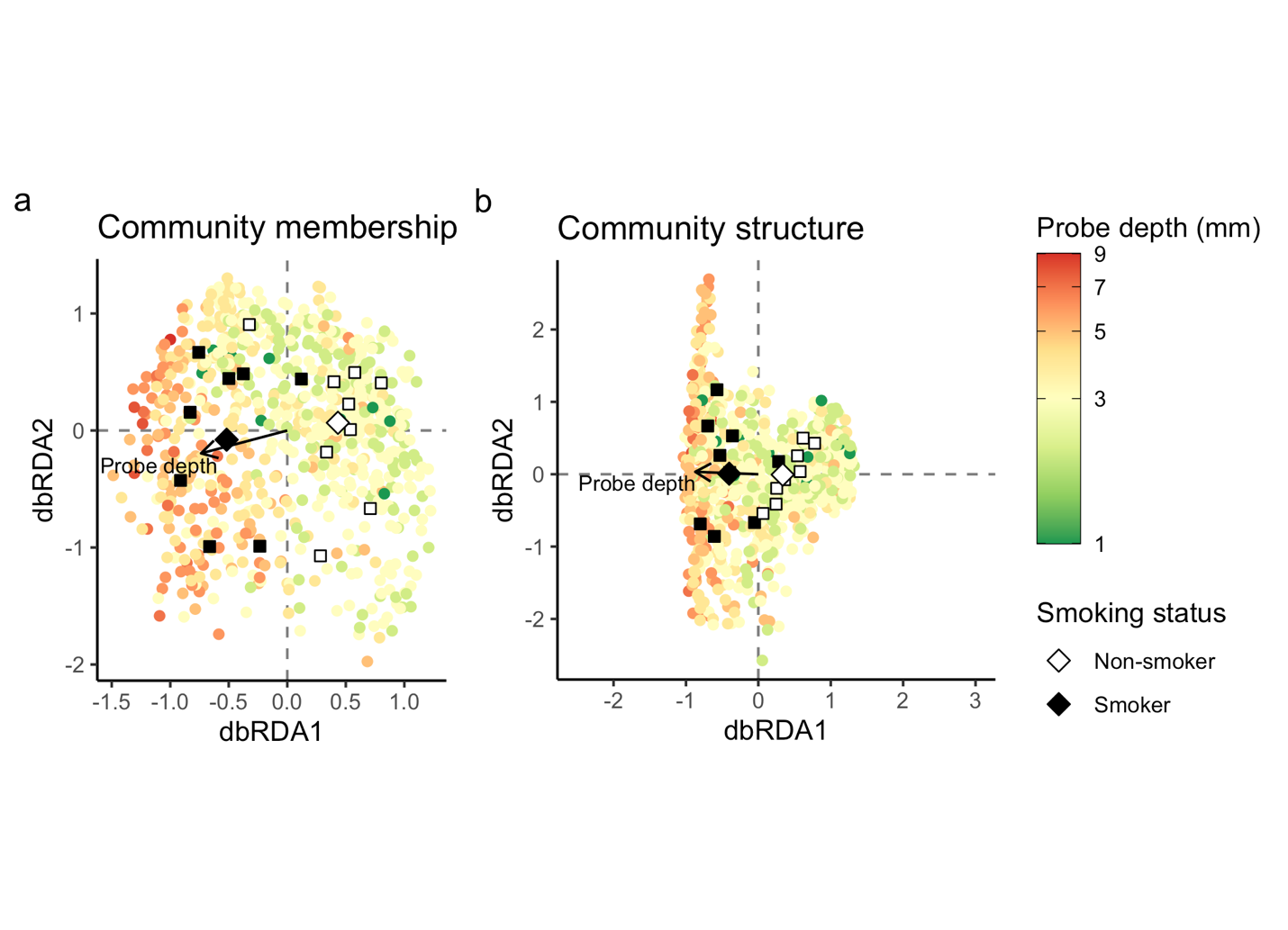
**Supplementary Figure 3**. Distance-based redundancy analysis (db-RDA) correlation biplot constrained by clinical and environmental variables.


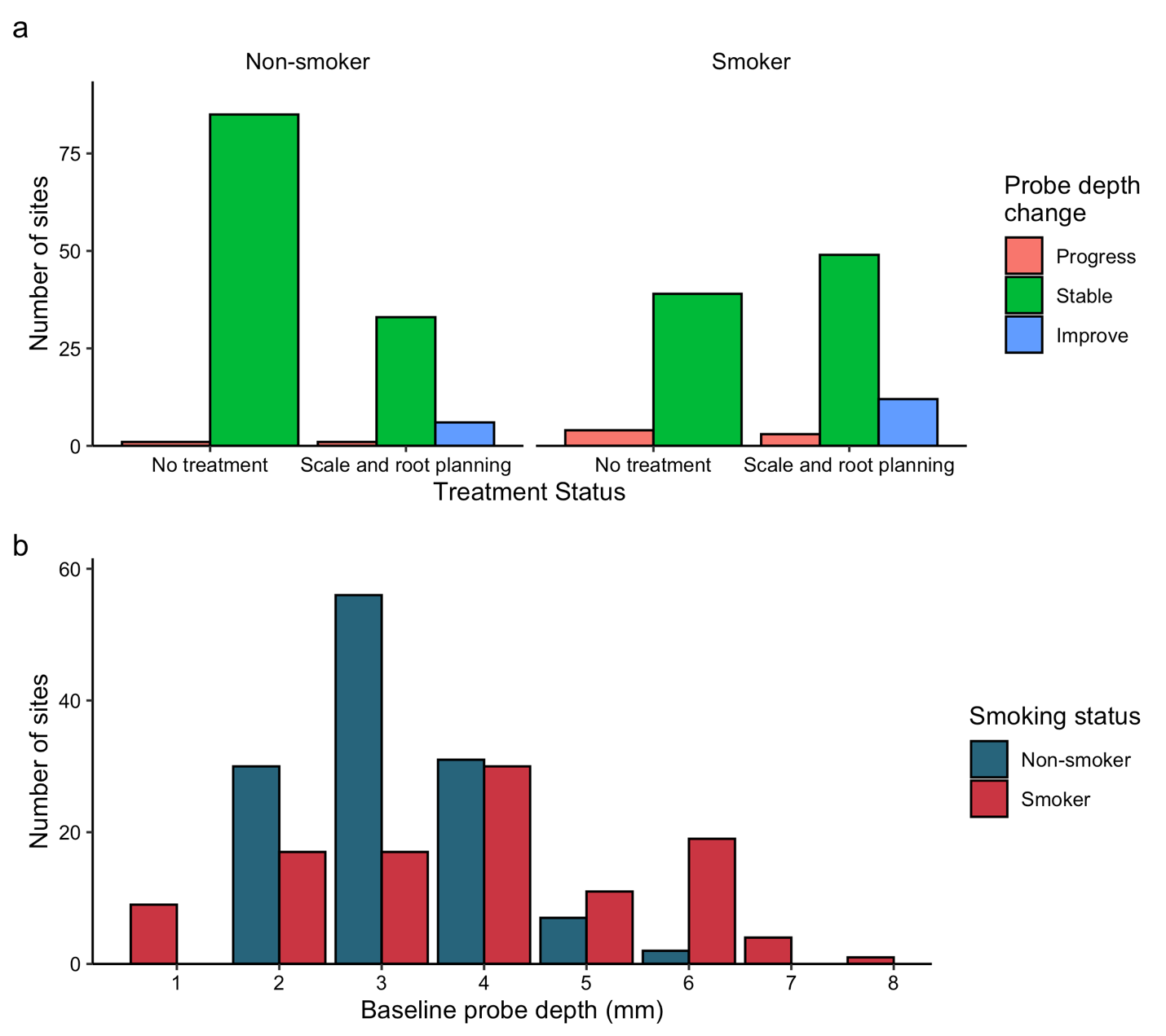


**Supplementary Figure 4.** Distribution of subgingival sites showing a) changes in probing depths (< 0 for improvement and ≥ 0 for no improvement) with regards to smoking status and treatment group and b) the initial probing depths.


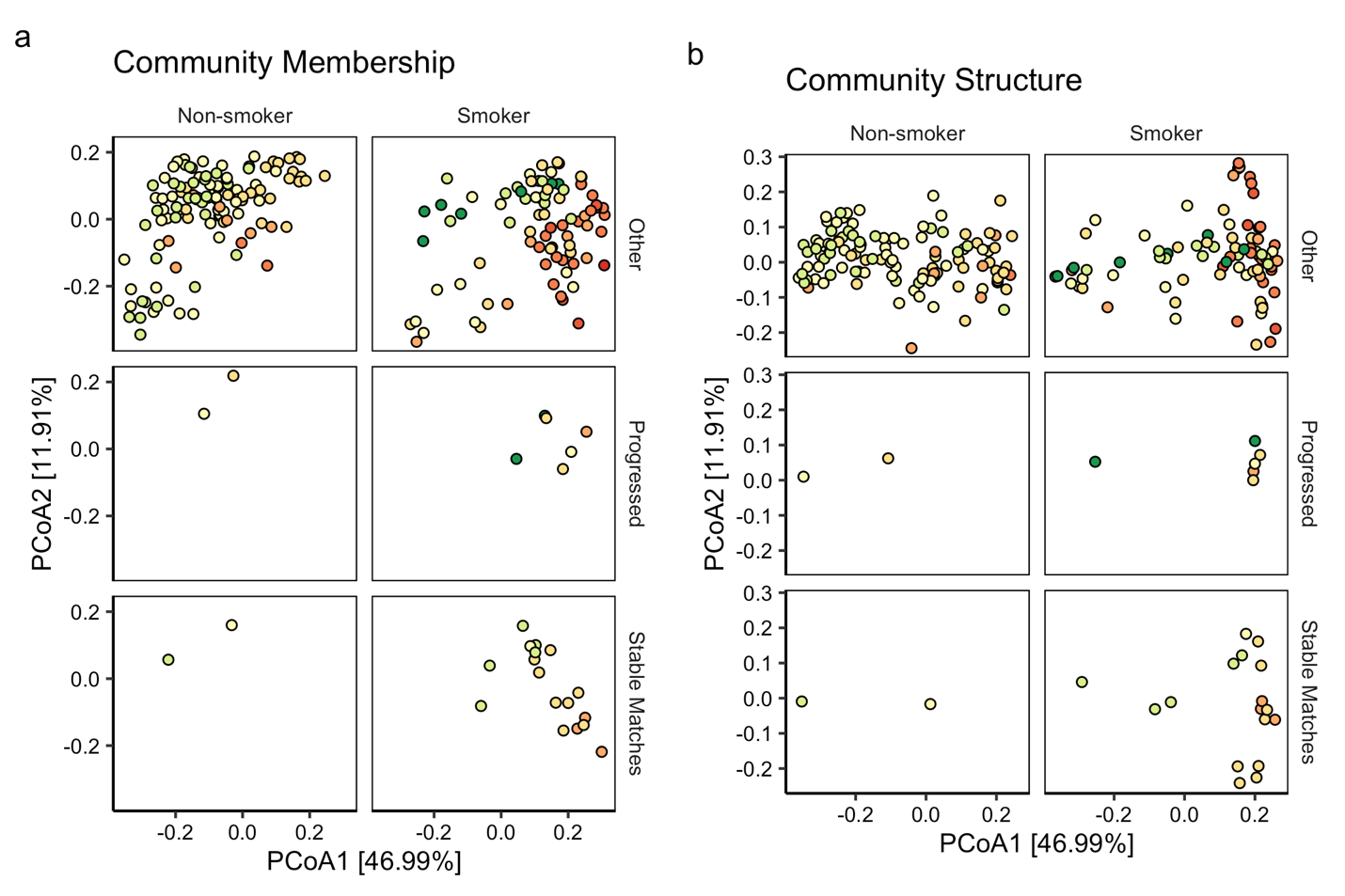


**Supplementary Figure 5.** Principal coordinates analysis on a) unweighted and b) weighted UniFrac distances between all baseline samples according to clinical progression or stability based on probing depths. In the middle panel, clinically progressed sites (change in probing depths of >1) clustered together near sites of deeper probing depths (top panel), whereas the stable matched sites (bottom panel) were more widely distributed.


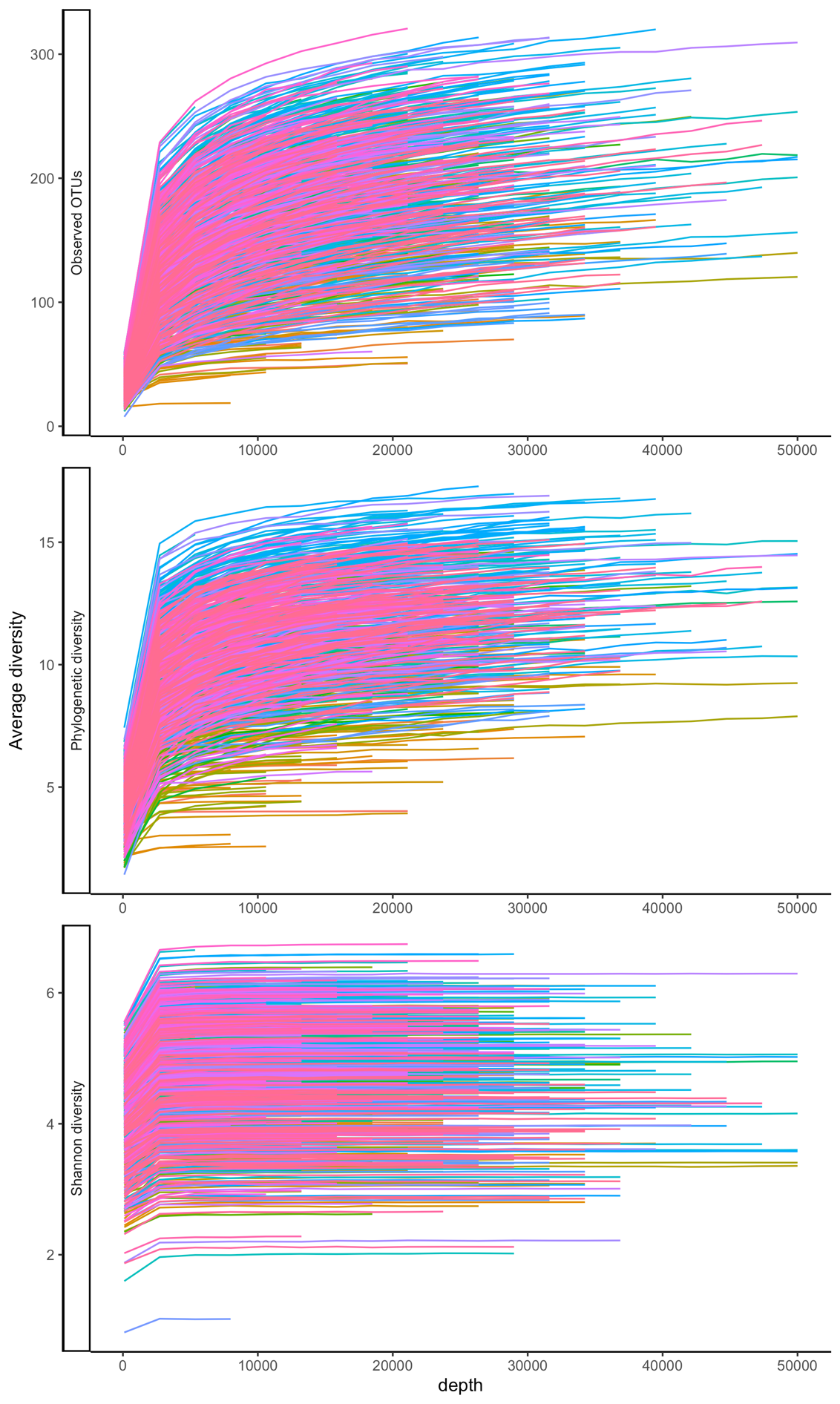


**Supplementary Figure 6**. Rarefaction analysis of the various diversity metrics by depth (in reads). Each line represents a unique sample.
